# Postharvest Quality of Parthenocarpic and Pollinated Cactus Pear [*Opuntia ficus-indica* L. (Mill)] Fruits

**DOI:** 10.3390/foods14142546

**Published:** 2025-07-21

**Authors:** Berenice Karina Flores-Hernández, Ma. de Lourdes Arévalo-Galarza, Manuel Livera-Muñoz, Cecilia Peña-Valdivia, Aída Martínez-Hernández, Guillermo Calderón-Zavala, Guadalupe Valdovinos-Ponce

**Affiliations:** 1 Colegio de Postgraduados, Campus Montecillo, Carretera México-Texcoco, Km. 36.5, Montecillo, Texcoco 56264, Mexico; flores.berenicekarina@colpos.mx (B.K.F.-H.); mlivera@colpos.mx (M.L.-M.); cecilia@colpos.mx (C.P.-V.); cazagu@colpos.mx (G.C.-Z.); gvapon@colpos.mx (G.V.-P.); 2 Colegio de Postgraduados, Campus Campeche, Carretera Haltún-Edzná, Km. 17.5, Champotón, Campeche 24450, Mexico; aida.martinez@colpos.mx

**Keywords:** nutraceutical, betacyanins, functional food, Cactaceae

## Abstract

*Opuntia ficus-indica* L. (Mill) belongs to the Cactaceae family. The plant produces edible and juicy fruits called cactus pear, recognized for their pleasant flavor and functional properties. However, the fruits have a short shelf life, hard seeds, and the presence of glochidia in the pericarpel. Recently, by inducing parthenocarpy, seedless fruits of cactus pear have been obtained. They have attractive colors, soft and small seminal residues, with a similar flavor to their original seeded counterparts. Nevertheless, their postharvest physiological behavior has not yet been documented. The aim of this study was to compare the biochemical, anatomical, and physiological characteristics of pollinated fruits, CP30 red and CP40 yellow varieties, with their parthenocarpic counterparts (CP30-P and CP40-P), obtained by the application of growth regulators in preanthesis. Fruits of each type were harvested at horticultural maturity, and analyses were carried out on both pulp and pericarpel (peel), using a completely randomized design. Results showed that red fruits CP30 and CP30-P showed higher concentrations of betacyanins in pulp (13.4 and 18.4 mg 100 g^−1^ FW) and in pericarpel (25.9 and 24.1 mg 100 g^−1^ FW), respectively; flavonoid content was significantly higher in partenocarpic fruits compared with the pollinated ones. Parthenocarpy mainly affected the shelf life, in pollinated fruits, CP30 was 14 days but 32 days in CP30-P; for CP40, it was 16 days, and 30 days in CP40-P. Also, the partenocarpic fruits were smaller but with a thicker pericarpel, and lower stomatal frequency. Overall, parthenocarpic fruits represent a viable alternative for commercial production due to their extended shelf life, lower weight loss, and soft but edible pericarpel.

## 1. Introduction

The genus *Opuntia* spp. is the largest of the Cactaceae family and includes a wide group of species and variants. The plant has modified stems (cladodes or nopales) that are consumed as vegetables and fleshy fruits called tunas or cactus pears. Mexico is the world’s leading producer of cactus pear, with a planted area of 45,033 ha, followed by Italy with 2.500 ha for fruit production [1,2].

The cactus pear is a simple berry, composed of sweet and juicy pulp that contains numerous seeds with a hard covering. The main pigments are betalains that present a wide variety of colors such as orange, red, or purple [3]. The fruit is formed from the lower ovary of a protandric, hermaphroditic flower whose differentiation period has been estimated at 35 to 70 days [4]. The time from anthesis to fruit maturity varies between 45 and 154 days depending on the species and variety, which allows them to be classified as early, medium, or late variants [5].

The fruits are rich in vitamins K (53.2–64.2 mg 100 g^−1^), C (28–79.2 mg 100 g^−1^) and E (527.4–623.2 mg 100 g^−1^), and minerals such as potassium (199–410.7 mg 100 g^−1^ DW), calcium (12.4–49.1 mg 100 g^−1^ DW) and magnesium (18 mg 100 g^−1^ DW). They are also sources of amino acids such as proline, taurine, serine, glutamine, leucine, lysine, and valine. Arginine, phenylalanine, and isoleucine, whose concentrations are between 2 and 35 mg 100 g^−1^. It also contains antioxidants such as polyphenols (protocatechuic acid, piscidic acid, caffeic acid 4-O-glucuronide, kaempferol-glucosyl rhamnoside, isorhamnetin glucosyl-rhamnoside, rutin, and eucomic acid) and high fiber content (4.65–5.83%) [6,7].

Mexico is the main consumer of tuna with 3.5 kg per capita per year, the green color variety “Reyna” is the most consumed, although there are varieties with attractive red or orange colors such as Roja San Martín and Roja Vigor (*O*. *ficus-indica*) and Naranjona (*O*. *megacantha*) [8], however 98% of the cactus pear production is for domestic consumption. The limitations of these fruits are their short shelf life (between 9 and 15 days at room temperature), sensitivity to chilling injury and the numerous hard seeds (20–30% of the total fruit), making them difficult to chew and with the possibility to be accumulated in the human appendix. Another constraint is that most fruit production is based on green color fruits, but now consumers prefer colors, such as red, purple, yellow, or orange. Therefore, characteristics such as appearance, resistance to postharvest handling, longer shelf life, and seedlessness in cactus pear would be an option for new markets [9].

Seed reduction or its elimination is a key objective in cactus pear breeding programs; however, the quality and fruit size often are correlated with seed development, representing a challenge [2]. To address this, growth regulators, principally gibberellic acid (GA_3_), have been used to induce parthenocarpy. Early studies demonstrated that applying GA_3_ (100–500 mg L^−1^) to emasculated flower buds produced seedless or seed-aborted fruits, although some had reduced pulp content and low soluble solids [10,11,12]. Similar results have been reported in *O. amyclaea*, where GA_3_ application produced small, underdeveloped fruits with aborted seeds [4]. Parthenocarpy was also induced in several Opuntia cultivars using 200 mg L^−1^ GA_3_, but the fruits were small [13]. The application (by injection and spraying) of gibberellic acid (100, 200, 250, and 500 mg L^−1^) in combination with flower bud emasculation was also evaluated in the Gialla variety. The spray treatment (500 mg L^−1^) was effective in reducing the number of seeds in the fruit by 46% [14].

The induction of parthenocarpic fruits is critically conditioned by the variety, as demonstrated by recent studies in tomato, where the response of mutant lines (pat-2, SlIAA9 RNAi, PROCERA) depends on the specific hormonal gene profile [15]. Then, in order to induce parthenocarpy in prickly pear, from 11 varieties, only two varieties, CP30 and CP40, produced parthenocarpic fruits of good quality based on size, color, and absence of seeds. The technique used is based on the removal of the sexual organs (style and stigma) of the floral along with the tepals and stamens, forming a cavity, then a solution of gibberellic acid (GA_3_; 250 mg L^−1^) + benzyladenine (BA; 75 mg L^−1^) + indole butyric acid (IBA; 15 mg L^−1^) was applied, avoiding runoff. The control (natural pollination) and an emasculated control without the application of growth regulators were also evaluated [16]. Based on the above, the objective of this research was to evaluate the postharvest quality characteristics of pollinated and parthenocarpic fruits.

## 2. Materials and Methods

### 2.1. Management and Description of Plant Material

**Figure 1:**
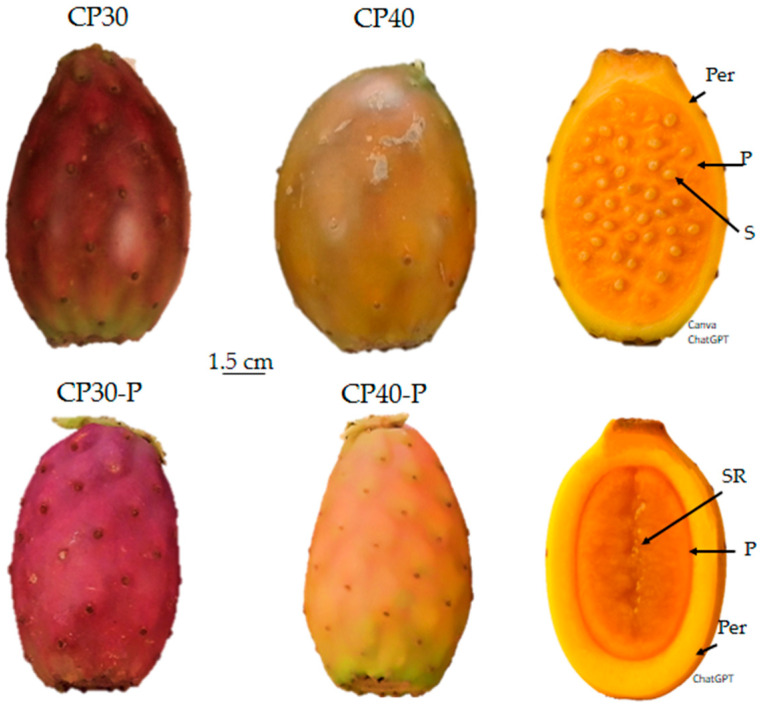
Appearance and longitudinal section of the prickly pear varieties (*O. ficus-indica* L. (Mill). CP30: Pollinated fruit, CP30-P: Parthenocarpic fruit, CP40: Pollinated fruit, CP40-P: Parthenocarpic fruit. Appearance and longitudinal section of the prickly pear varieties (*O. ficus-indica* L. (Mill). Per: pericarpel (peel); P: pulp; S: seed; SR: seminal residue. The descriptive image was generated with Canva (1.111.0) and ChatGPT (GPT-4o).

Prickly pear fruits of the CP30 red and CP40 yellow varieties pollinated and parthenocarpic ones were harvested from 7-year-old cactus pear plants from an experimental orchard in Mexico state, Mexico. Parthenocarpic fruits were obtained from emasculated flowers treated with a mix of growth regulators (250 mg L^−1^ GA_3_ + 75 mg L^−1^ BA + 15 mg L^−1^ IBA) at the preanthesis stage [16] (Figure 1).

A total of 130–200 fruits of each variety and condition were harvested at horticultural maturity (80% pigmentation) in July and August of 2023. A minimal fraction of the cladode was included with the fruit to prevent the peduncle rot. The gourds, or glochids, were removed with a self-made de-spinning machine, and the fruits were placed in plastic boxes and transferred to the laboratory.

The selected fruits were maintained at a temperature and humidity of 21 ± 1 °C and 61 ± 2% relative humidity for evaluation. The variables evaluated were shelf life, fruit weight, pulp/seed/pericarpel ratio, weight loss, stomatal frequency, pericarpel anatomy, color, titratable acidity, total soluble solids, soluble sugars, ascorbic acid, total phenols, total flavonoids, and antioxidant capacity. Pigments such as chlorophylls, carotenoids, betacyanins, and betaxanthins. Firmness and enzymatic activity of pectin methylesterase, polygalacturonase, catalase, and ascorbate peroxidase were determined. In addition, the content of some plant hormones in the fruits was evaluated. The evaluations were made at 0, 5, 10, and 15 days after harvest (dah) in CP30 and CP40, and at 0, 5, 10, 15, 20, 25, and 30 dah after CP30-P and CP40-P fruits. Catalase and ascorbate peroxidase were measured in CP30 and CP40 at 0 and 15 dah, and at 0, 15, and 30 dah in CP30-P and CP40-P.

### 2.2. Variables Evaluated

#### 2.2.1. Shelf Life

Ten fruits were kept under the aforementioned temperature and relative humidity conditions, and the shelf life ended when necrotic spots and signs of dehydration were evident.

#### 2.2.2. Fruit Weight, Pulp/Seed/Pericarpel Ratio, Weight Loss, and Fruit Size and Pericarp Thickness

Fruit weight (g) and pulp/seed/epidermis ratio (%) were determined in 10 fruits using a digital scale with a precision of 0.01 g (Ex2200 Alsep^®^, A&D Company, Tokyo, Japan). An additional 10 fruits were weighed every three days to evaluate weight loss, expressed as a percentage of the initial weight. In five fruits, fruit length and width were measured with a vernier caliper; to measure pericarpel thickness, the latter was removed from the pulp.

#### 2.2.3. Stomatal Frequency

Two impressions from the equatorial region of the epidermis were made in ten fruits, using the non-destructive microrelief technique with a dental resin (Xantopren^®^, Kulzer GmbH, Hanau, Alemania). The resin was applied on the epidermis, and upon drying, it was detached (negative impression). Transparent nail varnish was applied to this impression, and once the varnish layer was polymerized, it was peeled off (positive impression) and placed with the relief in reverse to its detachment on a slide. The positive was observed directly under an optical microscope (Olympus B50^®^, Olympus Corporation, Tokyo, Japan) [17]. The number of stomata was determined in each print, in five fields taken randomly at 150× magnification. Calculations were performed according to [18]. The area of the observed field was 2.14 mm^2^, obtained by measuring its diameter with a slide micrometer, visualized with the 10/0.25 lens of the optical microscope, and substituting the corresponding value in the formula and reported as the number of stomata mm^2^.

#### 2.2.4. Pericarpel Anatomy

The fruits were cut transversely, four pericarpel segments (0.5 cm wide) per fruit, per variety and condition were selected. These segments were placed in a solution (formaldehyde, glacial acetic acid, (96%) ethanol in the ratio 2:1:1, mixed with 350 mL of distilled water). Subsequently, the segments were washed with running water for 15 min and then dehydrated and infiltrated in an automatic tissue processor (Tissue-Tek^®^ II 4640-B^®^, Sakura Finetechnical Co., Ltd., Tokyo, Japan). After dehydration, the plant tissue was embedded in paraffin and cut transversely at 12 μm thickness on a microtome (Jung Histocut 820^®^, Leica brand, Wetzlar, Germany). The sections were mounted on slides with Haupt adhesive, spread on a hot plate (C. O. Slide 26020^®^, Thermo Scientific, Barnstead, IA, USA) at 20–25 °C for 24 h, and stained with safranin fast green [19]. In order to get the size, 35 parenchyma cells were measured. Observations were made using a optical microscope Axiostar Carl Zeiss^®^, Carl Zeiss Microscopy GmbH, Jena, Germany.

#### 2.2.5. Color

Color was determined on the equatorial section of five fruits using a colorimeter (NR110, 3nh^®^, Guangzhou, China). Values were expressed in L (lightness), C (chromaticity), and °H (hue angle).

#### 2.2.6. Titratable Acidity and Total Soluble Solids

Acidity was determined using the volumetric method [20] in 10 fruits. Ten g of tissue per fruit was weighed and liquefied with 50 mL of distilled water. A 5 mL aliquot of the mixture was taken, and three drops of phenolphthalein were added as a color indicator. The mixture was then titrated with NaOH (0.01 N) to pH 8. The results were expressed as % citric acid. Total soluble solids were determined in five fruits by weighing 5 g of the middle portion of each fruit, from which the juice was extracted, and placing two drops in a digital refractometer (PAL-1, ATAGO^®^, Tokyo, Japan). The result was expressed as °Brix [21].

#### 2.2.7. Soluble Sugars

The presence and concentration of sugars were determined in five fruits and quantified by high-performance liquid chromatography (HPLC) coupled to a refractive index detector (Perkin Elmer™, Series 200, Shelton, CT, USA). The evaluation was carried out using 3 g samples of tissue per fruit, finely chopped from the middle of the fruit. Alcoholic and solid-phase extraction (SPE) [22]. At 5 µm, a 150 × 4.6 mm Pinnacle II Amino column (Restek™, Bellefonte, PA, USA) was used. The mobile phase under isocratic conditions was acetonitrile/water (80:20, *v*/*v*) with a run time of 14 min. Calibration curves were prepared for fructose, glucose, and sucrose (99.5%, Sigma–Aldrich, St. Louis, MO, USA). The chromatograph conditions were as follows: column temperature: 35 °C, flow rate: 1 mL min^−1^, and injection volume: 10 µL. Results were expressed in g ± 100 g^−1^ fresh weight (FW).

#### 2.2.8. Ascorbic Acid

Ascorbic acid concentration was determined using the 2,6-dichlorophenol-indophenol method [20]. Two grams of tissue per fruit were taken and homogenized with 20 mL of oxalic acid (5%). The fruit was then titrated with Tillman’s solution until a pink color was obtained. The results were expressed in mg 100 g^−1^.

#### 2.2.9. Total Phenols

Total phenol concentrations were quantified using 1 N Folin–Ciocalteu reagent (Sigma–Aldrich, St. Louis, MO, USA) [23]. Quantification was performed on five fruits, and one gram of tissue was weighed from each fruit to obtain an 80% methanol extract. Samples were read at 765 nm using a spectrophotometer (Genesys 10 UV-Vis, Thermo Spectronic^®^, Madison, WI, USA), and absorbances were interpolated into the equation obtained from the gallic acid calibration curve. Results were expressed as milligrams of gallic acid equivalent per gram of fresh weight (mg EGA 100 g^−1^ FW).

#### 2.2.10. Total Flavonoids

This was determined based on the modified colorimetric method [24]. Five fruits were quantified, and a methanolic extract was made from each fruit; 500 μL of the diluted extract was mixed with 2 mL of methanol and 200 μL of a 2% AlCl_3_ solution. After incubating for 3 min at room temperature, 200 μL of 1 M CH_3_COONa was added to the mixture. The final volume was adjusted to 5 mL with methanol. After 40 min of incubation in the dark at room temperature, the absorbance was measured at 430 nm. A parallel calibration curve was created under the same operating conditions using quercetin. The flavonoid content was expressed in milligrams of quercetin equivalent per gram of dry weight (mg QE 100 g^−1^ DW).

#### 2.2.11. Antioxidant Capacity

Antioxidant capacity was evaluated following the method, including 2,2-diphenyl-1-picrylhydrazyl (DPPH) (Sigma–Aldrich; St. Louis, MO, USA) with some modifications [25]. This was determined in five fruits. The violet colored DPPH turns yellowish upon contact with the antioxidant sample, and readings were taken at 517 nm on a spectrophotometer (Genesys 10 UV-Vis, Thermo Spectronic^®^, Madison, WI, USA)) at 10, 20, 30, and 60 min. Antioxidant capacity was represented as radical scavenging capacity (% RSC) with the absorbance of DPPH without sample at time zero being 100%.

#### 2.2.12. Chlorophyll and Carotenoids

Chlorophyll and carotenoid concentrations were determined according to the method described by Wellburn (1994) [26]. Tissue (0.1 g) from each fruit was macerated with 10 mL of acetone (80%) (Sigma–Aldrich; St. Louis, MO, USA) and placed in polypropylene tubes. The extract was centrifuged at 4000× *g* for 20 min, and then the absorbances were read at 470, 646, and 663 nm, respectively, in a spectrophotometer (Genesys 10 UV-Vis^®^, Thermo Spectronic, Madison, WI, USA)). Chlorophyll and carotenoid contents were calculated using the formulas reported by Lichtenthaler [27] and expressed as mg 100 g^−1^.

#### 2.2.13. Betalains (Betacyanins and Betaxanthins)

Betalains were determined in five fruits; samples of 2 g of tissue were weighed and macerated with 20 mL of methanol/water (80:2, *v*/*v*), then stirred for 45 min in the dark. After centrifugation at 4000× *g* for 10 min, the supernatant was collected. The process was repeated with the residue, but with 10 mL of methanol/water (80:20, *v*/*v*). Finally, the extracts were combined, and the absorbance was read at 483 and 538 nm, respectively, in a spectrophotometer (Genesys 10 UV-Vis^®^, Thermo Spectronic, Madison, WI, USA). To calculate the concentration, the equation cited by García-Cruz et al. [28] was used.

#### 2.2.14. Firmness

Firmness was analyzed in five fruits, each measured using a universal texturometer (Force Five FDV-30, WAGNER^®^, Greenwich, CT, USA) equipped with a 7 mm diameter conical probe. The texturometer measures the force required to penetrate first the pericarpel and then the pulp without pericarpel in whole fruits. Values are expressed in Newtons (N).

#### 2.2.15. Pectinmethyl Esterase (PME)

A standard 1% (*w*/*v*) pectin solution (Sigma-Aldrich^®^, Burlington, MA, USA) was prepared (pH = 2.8–3.5) and adjusted to 4 with NaOH (1 N) (JT BAKER^®^; Madrid, Spain). Subsequently, the potentiometer was placed in the solution, 0.8 mL of enzyme extract (20 g of tissue + 50 mL of NaOH (0.2 N)) was added, and the time was recorded. During the analysis, the pH was maintained at 4 with NaOH (0.01 N) for 10 min at 40 °C. The mL of NaOH used was quantified, and the results were expressed in meq mL^−1^ min^−1^ [29]. PME activity was measured in the pulp and pericarpel of five fruits.

#### 2.2.16. Polygalacturonase (PG)

PG activity was evaluated with 3,5-dinitrosalicylic acid (DNS) [30] and was analyzed in the pulp and pericarpel of five fruits. PG activity was measured using Miller’s reagent (3,5-dinitrosalicylic acid) (JT BAKER^®^, Madrid, Spain). The reaction between the released reducing terminal and 3,5-DNS acid was obtained. The absorbance of the samples was determined at 540 nm in a spectrophotometer (Genesys 10 UV-Vis^®^, Thermo Spectronic, Madison, WI, USA). PG activity was calculated by interpolating the readings into a galacturonic acid curve (Sigma-Aldrich^®^, Burlington, MA, USA), and the result was expressed as U g^−1^ FW.

#### 2.2.17. Catalase (CAT)

Acetone powder was prepared from five fruits; for this, 10 g of pericarpel (1 fruit as a replicate) was crushed with cold acetone, repeated this action three times, then dried. Afterwards, the powder obtained was stored in the freezer. The CAT analysis was measured by taking 0.1 g of acetone powder, mixing it with 5 mL of TRIS-HCl buffer (0.1 M, pH 8.5) and 2% PVP, and centrifuging at 4000× *g* at 4 °C for 30 min. Then, 200 μL of the supernatant was mixed with 200 μL of H_2_O_2_ (0.2%) and 2 mL of TRIS-HCl buffer (10 mM, pH 8.5). The absorbance was recorded every 10 s for one minute at 240 nm in a spectrophotometer (Genesys 10s UV-Vis^®^, Thermo Spectronic, Madison, WI, USA) [31]. The results are expressed in U g^−1^ FW.

#### 2.2.18. Ascorbate Peroxidase(APX)

APX activity was measured by taking 0.1 g of acetone powder, adding 5 mL of phosphate buffer (50 mM, pH 7.8) plus 0.2 mM EDTA (ethylenediaminetetraacetic acid) (Sigma-Aldrich, São Paulo, Brazil) and 2% PVP (polyvinylpyrrolidone) (Sigma-Aldrich, São Paulo, Brazil). The samples were centrifuged at 4000× *g* at 4 °C for 30 min, then 200 μL of the supernatant was taken and mixed with 200 μL of H_2_O_2_ (0.2%) and 2 mL of phosphate buffer (50 mM, pH 7.0) [32]. The absorbance was recorded every 10 s for one minute at 290 nm in a spectrophotometer (Genesys 10s UV-Vis^®^, Thermo Spectronic, Madison, WI, USA). The results are expressed in U g^−1^ FW.

#### 2.2.19. Determination of Endogenous Plant Hormones

Previously frozen pericarpel and pulp samples were placed in a freeze-dryer (Labconco) for 72 h, with three samples per tissue and variety. Hormone extraction was carried out using the method proposed by Pan et al. [33]. Endogenous hormone analysis and quantification were performed using a diode array detector; 100 mg samples were placed in plastic tubes (2.0 mL), and 500 µL of extraction solution (2-propanol:water: concentrated HCl (2:1:0.002, *v*/*v*)) was added. Tubes were shaken at 100 rpm for 30 min at 4 °C, then 1 mL of methylene chloride HPLC grade was added and stirred for another 30 min. The 900 µL of supernatant was transferred to an amber vial and dried under a stream of nitrogen gas until the volume reached 100 µL. Following that, 500 µL of methanol, HPLC grade, was added. A 100 µL of the sample was injected into the liquid chromatograph HPLC. The liquid chromatograph (Agilent 1100, Agilent Technologies^®^, Santa Clara, CA, USA) was equipped with an automatic injector model 1200 and a model 1100 Diode Array Detector, Agilent Technologies (Santa Clara, CA, USA) Rx/SB-C8 Rapid Res 4.6 × 75 column, with the mobile phase consisting of the following solvents: A (80%): 0.1% trifluoroacetic acid and B (20%): 0.1% trifluoroacetic acid in acetonitrile. The flow was 2 mL min^−1^, the temperature was 60 °C, the injection volume was 100 µL, and the detector was set at 254 nm. The hormonal profile involved the identification of some important hormones in their free forms. Indoleacetic acid (IAA) and indole-3-butyric acid (IBA) were determined, the latter with little known functions in plants and little studied in fruits. Gibberellic acid (GA_3_) and abscisic acid (ABA) were also evaluated. Each endogenous hormone was identified and quantified using a standard curve, according to growth regulatory standards (Sigma-Aldrich; Saint Louis, MO, USA). One fruit per treatment was a replicate; three replicates were performed for this analysis.

### 2.3. Statistical Analysis

This study was developed under a completely randomized design where the fruits of each variety were the treatments, and each replicate consisted of one fruit. CP30 and CP30-P fruits were compared with each other, and the same was conducted for CP40 and CP40-P fruits. Data were expressed as mean ± standard deviation, and the analysis of variance (ANOVA) and Tukey’s test (α = 0.05) were also performed. All analyses were performed with SAS software (On Demand for Academics^®^ version 9.04) [34], and graphs were obtained with GraphPad Prism software version 7.00.

## 3. Results

### 3.1. Shelf Life

**Figure 2:**
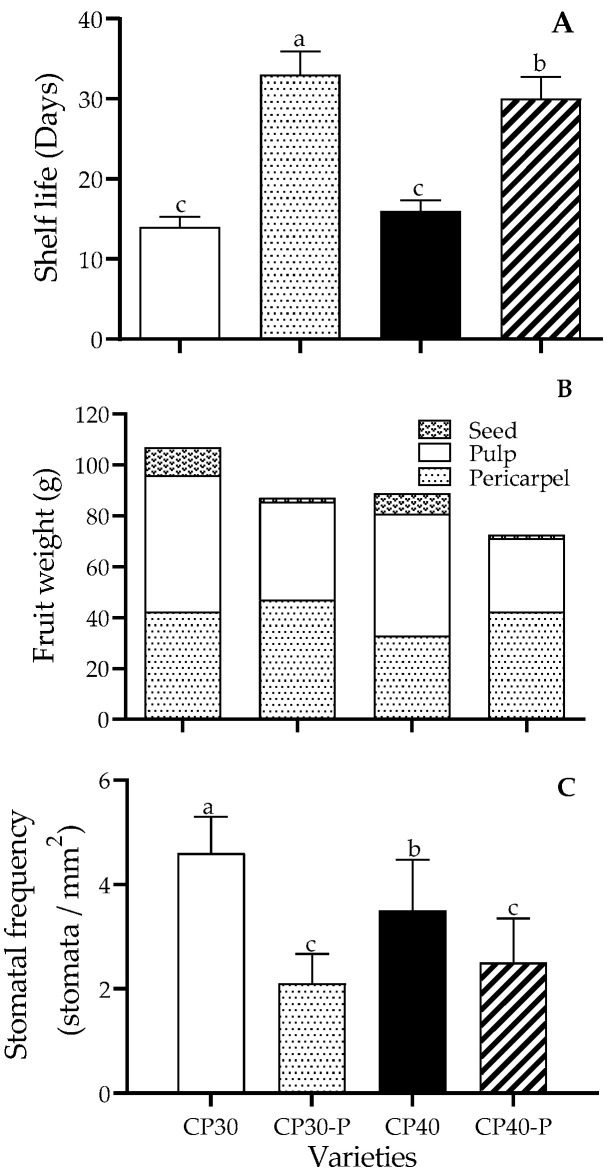
(**A**) Shelf life (*n* =10 ± SD), (**B**) Weight and pulp/seed/pericarpel ratio (*n* = 10) and (**C**) stomatal frequency in the epidermis (*n* = 10 within five fields each ± SD) of prickly pear fruits (*O. ficus-indica* L. (Mill)), maintained at 21 ± 1 °C and 61 ± 2% RH. Values with different letters indicate statistically significant differences (Tukey, *p* ≤ 0.05) between each variety. CP30: Pollinated; CP30-P: Parthenocarpic, CP40: Pollinated, CP40-P: Parthenocarpic.

The shelf life in CP30 was 14 days and 32 days in CP30-P, for CP40 was 16 days, and 30 days in CP40-P (Figure 2A). Shelf life was more than doubled in parthenocarpic fruits, representing a significant advantage for their postharvest handling.

### 3.2. Fruit Weight, Size, and Pulp/Seed/Pericarpel Ratio

The parthenocarpic fruits were smaller than the pollinated ones; however, seeds were approximately 10% of the total weight in these fruits, compared to only 2% in parthenocarpic ones (Figure 2B). Pollinated fruits were wider (4.5 ± 0.2 cm) than parthenocarpic fruits (4.0 ± 0.1 cm); however, their pericarpel was significantly softer, but thicker (0.6 ± 0.08 cm) compared to that of pollinated fruits (0.3 ± 0.09 cm), making them more suitable for consumption. Therefore, if these fruits were marketed on a larger scale, there would be advantages since, to obtain 1 kg of edible portion, 17 pollinated fruits are required, but only 12 parthenocarpic fruits are needed.

### 3.3. Stomatal Frequency

The stomatal frequency of the fruits was significantly higher in pollinated CP30 and CP40 fruits than in parthenocarpic CP30-P and CP40-P, with a 2:1 ratio (Figure 2C), which could allow for lower weight loss in the latter.

### 3.4. Pericarpel Anatomy

**Figure 3:**
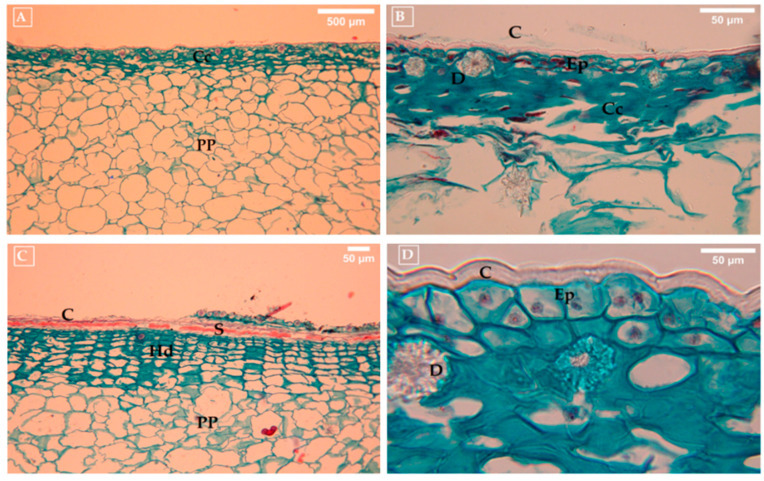
(**A**) Histological sections of the pericarpel of pollinated fruits, (**B**) Sections of the epidermis of pollinated fruits (**C**) Histological sections of the pericarpel of parthenocarpic fruits, (**D**) Sections of the epidermis of the pericarpel of parthenocarpic fruits of prickly pear (*O. ficus-indica* L. (Mill)). C: cuticle, S: sclereids; PP: pericarpel parenchyma; D: drusen; Ep: epidermis; Cc: collenchyma; Hd: hypodermis.

Structural and quantitative differences were observed between parthenocarpic and pollinated fruits; pollinated fruits lacked sclereids and hypodermis, but showed a higher density of smaller parenchymal cells, and average the cell area of 0.282 µM (Figure 3A), with a less organized structure, thinner cuticle (0.059 µM) and the presence of collenchyma (Figure 3B). Drusen were observed in all types of fruits (Figure 3B,D). The pericarpel of parthenocarpic fruits presents a well-defined row of sclereids and a distinctive hypodermal layer (Figure 3C). These characteristics could explain the easy separation of the epidermis from the pericarpel. Furthermore, the parenchymal tissue exhibited an orderly cellular arrangement and larger cell size, with an area of 0.740 µm (Figure 3C), and a thicker cuticle (0.161 µm) (Figure 3D).

### 3.5. Weight Loss

The pollinated fruits had a shelf life of 14 days for CP30 and 16 days for CP40; the parthenocarpic ones had 32 days for CP30-P and 30 days for CP40-P, when fruits had weight losses around 10% (Table 1).

### 3.6. Color of Pulp and Pericarpel

The parthenocarpy did not affect the color of the fruits since no significant statistical differences were observed. The red pulp in fruits CP-30 and CP30-P had values of L* = 43, and in pericarpel 54. In Chroma*, the values were 54 and 49, respectively; Hue* values were 44 in pulp and 35 in pericarpel. Regarding the yellow ones (CP-40 and CP40-P), the L* value in pulp and pericarpel was 68, for Chroma* values of 39 and 64, for Hue* values of 65 and 78, respectively. Therefore, the fruits could have acceptance and demand in different markets due to their color, compared to most commercial green prickly pear fruits (Figure 1).

### 3.7. Total Soluble Solids(TSS), Acidity(A), TSS/TA Ratio and Soluble Sugars

No significant differences were found in total soluble solids (TSS) between CP30, CP40, and their parthenocarpic counterparts, with values ranging from 12.7 to 15.5 °Brix. Similarly, titratable acidity ranged from 0.19 to 0.30% in all varieties. The TSS/acidity (TSS/A) ratio also showed no differences, ranging from 48 to 75. Glucose and fructose were the main sugars, with higher concentrations in the pulp than in the pericarpel, and no significant differences between parthenocarpic and pollinated fruits. On average, CP30 and CP30-P showed 2.2% fructose and 3.7% glucose in the pericarpel, and 3.9% fructose and 7.9% glucose in the pulp. For CP40 and CP40-P, the content of fructose was 2.5% and for glucose, 3.6% in the pericarpel, while the pulp contained 4.7% fructose and 8.3% glucose.

### 3.8. Ascorbic Acid

No significant differences in ascorbic acid content among varieties or conditions were found. On average, at day 0, the content in pericarpel was 57 mg 100 g^−1^ FW and in the pulp, 62 mg 100 g^−1^ FW. At day 10 of storage, the pericarpel had 39 mg and the pulp had 55 mg 100 g^−1^ FW. The degradation was higher (31%) in the pericarpel compared with the pulp (11%) after 10 days of storage.

### 3.9. Total Phenols and Antioxidant Activity

The pulp of CP30 had a significantly higher content of phenols compared with CP30-P in CP40 fruit; no differences were found. After 10 days of storage, the reduction of total phenols is very dramatic, except in the pericarpel of CP30-P. Related to the antioxidant activity, there are no differences between pollinated and parthenocarpic fruits (Table 2).

### 3.10. Total Flavonoid

Total flavonoid concentrations differed significantly between parthenocarpic and non-parthenocarpic varieties. On day 0, CP30-P and CP40-P fruits exhibited higher flavonoid levels in the pericarpel compared to their pollinated counterparts. After 10 days of storage, all varieties showed an increase in flavonoids. On average, the concentration in the pericarpel was four times higher than in the pulp; CP40 and CP40-P fruits had the highest levels. Although CP40 fruits were higher on day 10, it is worth noting that the pericarpel of parthenocarpic prickly pears is edible, and its high flavonoid content represents a clear advantage in terms of nutritional value and market potential (Table 3).

### 3.11. Pigments (Chlorophyll, Carotenoids, and Betalains)

Prickly pear fruits contain 2 to 3 mg 100 g^−1^ fresh weight (FW) of chlorophyll in the pericarpel and very little in the pulp. The carotenoid content was higher in the pollinated varieties than in the parthenocarpic ones, being more abundant in the pericarpel of CP40. However, while the pericarp of pollinated fruits is not edible, it is edible in parthenocarpic fruits. In the pulp of CP30 fruits, it was 40% higher than in CP30-P fruits, with significant differences between varieties of the same color (Table 4). The concentration of betacyanins in red CP30 and CP30-P fruits is significantly higher than in yellow fruits (CP40 and CP40-P), which explains their color, although without significant differences (Table 5).

### 3.12. Firmness

**Figure 4:**
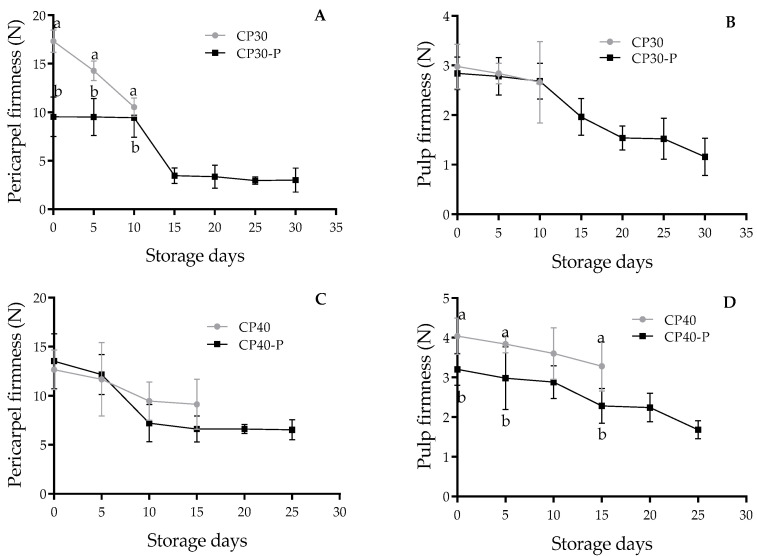
Changes in firmness of prickly pear fruits (*O. ficus-indica* L. (Mill)), stored at 21 ± 1 °C and 61 ± 2% RH. (**A**) Pericarpel firmness of CP30 and CP30-P, (**B**) Pulp firmness of CP30 and CP30-P, (**C**) Pericarpel firmness of CP40 and CP40-P, (**D**) Pulp firmness of CP40 and CP40-P. Values with different letters indicate statistically significant differences (Tukey, *p* ≤ 0.05) for each variety. CP30: Pollinated fruits, CP30-P: Parthenocarpic, CP40: Pollinated fruits, CP40-P: Parthenocarpic (*n* = 5 ± SD).

The pericarpel of CP30 fruits presented greater firmness (17.32 N) than CP30-P (9.52 N); however, after ten days of storage, firmness decreased by 40%. The parthenocarpic fruits remained firm, and from day zero to day ten of storage, they lost only 0.89% (Figure 4A). In CP40 and CP40-P fruits, the behavior was similar from harvest to day five (13 N); and after ten days of storage, the drop in firmness was abrupt, since pollinated fruits lost 0.24 N per day, compared to parthenocarpic fruits that lost 0.28 table per day (Figure 4C). Regarding the pulp of CP30 and CP30-P fruits, no significant differences in pulp firmness were observed; however, significant differences were found between the pulp firmness of CP40 and CP40-P fruits (Figure 4B,D).

### 3.13. Pectinmethyl Esterase (PME)

**Figure 5:**
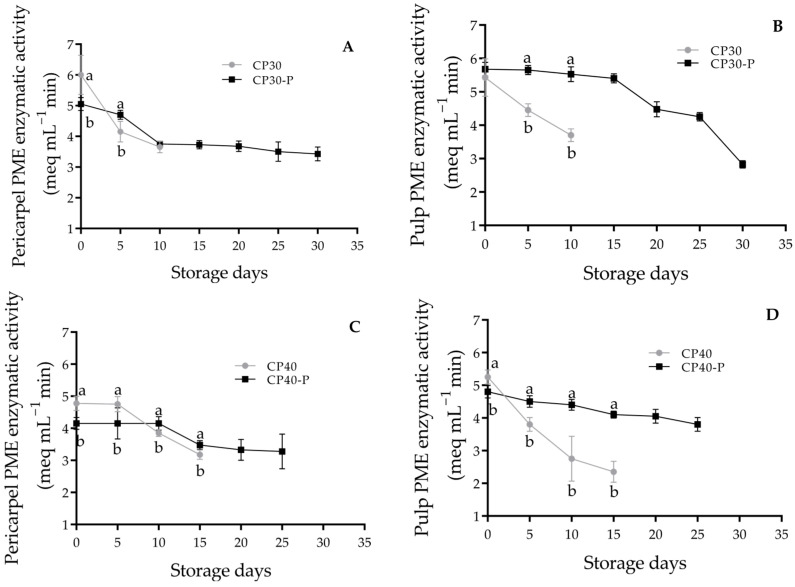
Pectinmethylesterase activity of prickly pear fruits (*O. ficus-indica* L. (Mill)), stored at 21 ± 1 °C and 61 ± 2% RH. (**A**) Pericarpel PME enzymatic activity of CP30 and CP30-P, (**B**) Pulp PME enzymatic activity of CP30 and CP30-P, (**C**) Pericarpel PME enzymatic activity of CP40 and CP40-P, (**D**) Pulp PME enzymatic activity of CP40 and CP40-P. Values with different letters indicate statistically significant differences (Tukey, *p* ≤ 0.05) in each variety. CP30: Pollinated fruits, CP30-P: Parthenocarpic, CP40: Pollinated, CP40-P: Parthenocarpic (*n* = 5 ± SD).

PME enzyme activity in the pericarpel of CP30 fruits at day 0 was higher than that of the CP30-P fruits, however, a drastic reduction was observed at the end of its shelf life (15 days) with 3.65 meq mL^−1^ min^−1^. In contrast, parthenocarpic fruits maintained an activity of 5.05 to 3.75 meq mL^−1^ min^−1^ until the end of storage at day 30 (Figure 5A). In the pericarpel of CP40 fruits, PME activity showed a continuous decline after the fifth day of storage. For CP40-P fruits, the reduction in activity by day 10 was not significant, with values remaining around 3.36 meq mL^−1^ min^−1^ (Figure 5C). Additionally, PME activity in the pulp of CP40-P fruits was significantly higher (4.37 meq mL^−1^ min^−1^) and stable during the storage period (Figure 5D).

### 3.14. Polygalacturonase (PG)

**Figure 6:**
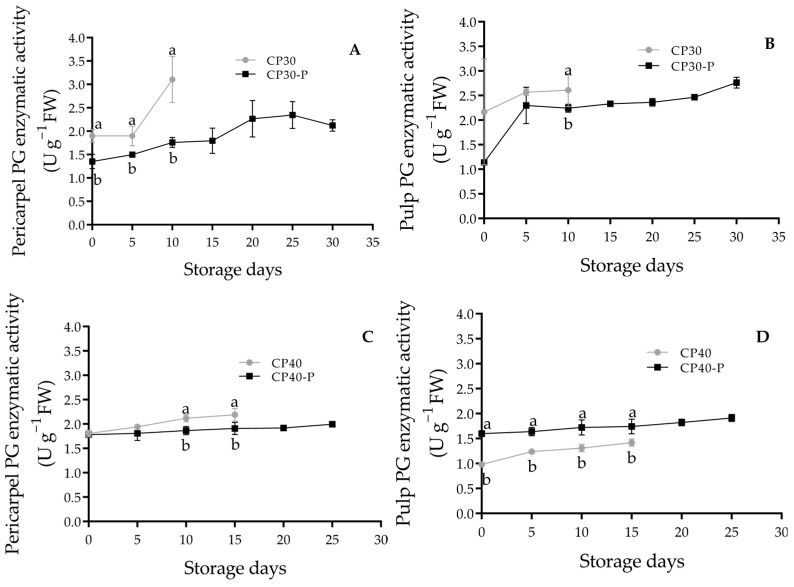
Polygalacturonase activity of prickly pear fruits (*O. ficus-indica* L. (Mill)), stored at 21 ± 1 °C and 61 ± 2% RH. (**A**) Pericarpel PG enzymatic activity of CP30 and CP30-P, (**B**) Pulp PG enzymatic activity of CP30 and CP30-P, (**C**) Pericarpel PG enzymatic activity of CP40 and CP40-P, (**D**) Pulp PG enzymatic activity of CP40 and CP40-P. Values with different letters indicate statistically significant differences (Tukey, *p* ≤ 0.05) in each variety. CP30: Pollinated fruits, CP30-P: Parthenocarpic, CP40: Pollinated fruits, CP40-P: Parthenocarpic (*n* = 5 ± SD).

The pericarpel of CP30 fruits showed a 43% higher activity compared to CP30-P (Figure 6A). The same trend was observed in CP40 fruits, which had a PG activity 12% higher than CP40-P at the beginning of the storage (Figure 6C). Related to the pulp, the CP40 fruits showed lower enzymatic activity in PME and PG, and higher firmness than the CP40-P fruits.

### 3.15. Catalase (CAT) y Ascorbate Peroxidase (APX)

Catalase (CAT) and ascorbate peroxidase (APX) showed significant variations between varieties and during storage. At 0 days of storage, differences were only observed in yellow fruits regarding CAT, while for APX, it was observed in CP30 red fruits. After 15 days of storage, a notable increase in CAT activity was observed in all varieties. CP30-P registered the highest activity (1495.0 ± 72.4 U g^−1^ FW), followed by CP40-P, which presented on average 41% higher activity than pollinated fruits. At 30 days of storage, the activity of both enzymes decreased by 10% in parthenocarpic fruits (Table 6).

### 3.16. Plant Hormone Content

**Figure 7:**
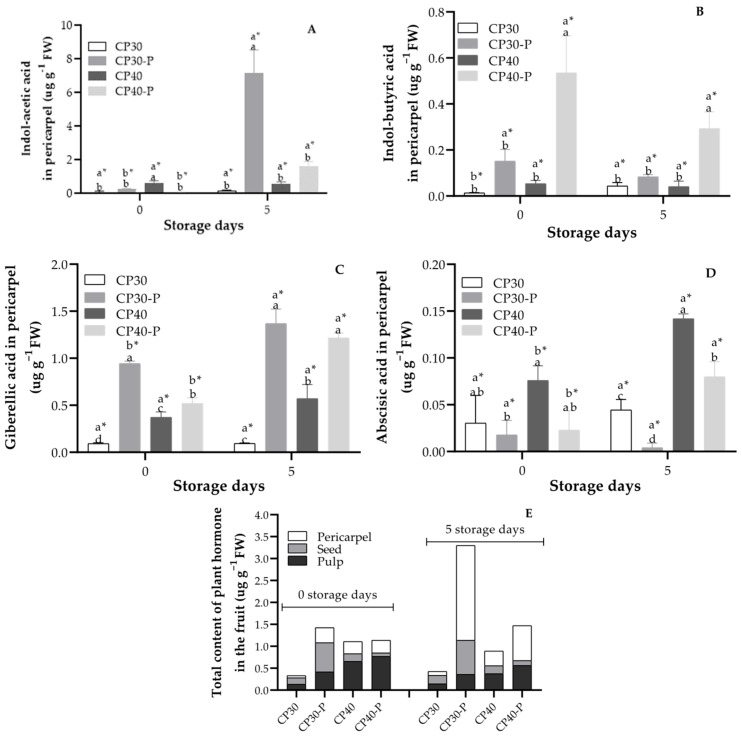
Content of plant hormone of prickly pear fruits (*O. ficus-indica* L. (Mill)), stored at 21 ± 1 °C and 61 ± 2% RH. (**A**) Indole-acetic acid content in pericarpel, (**B**) Indol-butyric acid content in pericarpel, (**C**) Giberellic acid content in pericarpel, (**D**) Abscisic acid content in pericarpel, (**E**) Total content of plant hormone in the fruit. * Values with different letters indicate statistically significant differences (Tukey, *p* ≤ 0.05) for the same fruit type on different days. In the second row, different letters indicate statistically significant differences (Tukey, *p* ≤ 0.05) among fruit types within each day. CP30: Pollinated; CP30-P: Parthenocarpic, CP40: Pollinated, CP40-P: Parthenocarpic (*n* = 3 ± SD).

During storage, significant differences were observed in the levels of endogenous growth regulators. On day 5, the pericarpel of CP30-P fruits had a markedly higher indoleacetic acid (IAA) concentration (7.18 µg g^−1^ FW) compared to CP-30 (0.18 µg g^−1^ FW), making IAA the most abundant hormone among those evaluated (Figure 7A). Indole-3-butyric acid (IBA) showed its highest concentration at day 0 in CP40-P fruits but declined by day 5 (Figure 7B). Gibberellic acid (GA_3_) content increased throughout storage, rising by 43% in parthenocarpic fruits between day 0 and day 5, while the increase was lower in pollinated fruits (Figure 7C). In contrast, abscisic acid (ABA) levels were significantly higher in pollinated fruits at day 5, especially when compared to CP30-P fruits, which had the lowest ABA concentration (0.04 µg g^−1^ FW), suggesting a link between ABA and shelf life (Figure 7D). Overall, the total hormone content in the pericarpel peaked at day 5, with the most substantial increase (84%) observed in CP30-P fruits, correlating with their longer postharvest life (Figure 7E).

## 4. Discussion

Parthenocarpic fruits represent an advantage since fruits with fewer or abortive seeds are more appreciated by consumers [35]. Furthermore, the large number of seeds in pollinated fruits complicates their industrial processes [36]. One of the main advantages that parthenocarpic fruits have is the higher edible part (pericarpel and pulp). In *O. ficus-indica* L. (Mill) cv. “Gialla”, a pericarpel 50% thicker than pollinated fruits, was observed [14]. In *O. ficus-indica* L. (Mill) var. Amarilla 3389 and Naranjona, induced with gibberellin, the thickness of the pericarpel was 0.9 cm compared to normal fruits (0.5 cm) [37]. This characteristic has also been observed in parthenocarpic tomato (*Solanum lycopersicum* Will.), where auxin-regulated cell division (phase II of cell division) is omitted, while phase III of cell expansion is activated only in the epidermal and hypodermal tissue, so this may cause a thicker pericarp [38,39]. In parthenocarpic fruits of *Opuntia,* certain gibberellin biosynthesis genes that contribute to fruit development are activated. However, the dual involvement of the funicles in the development of the testa and pulp, which occurs during phase II (cell division and seed formation), is halted, while phase III of growth (cell expansion) remains active [40].

Parthenocarpic fruits have a longer shelf life, probably due to the endogenous content of growth regulators derived from their application at preanthesis [14]. This characteristic has also been observed in parthenocarpic tomato fruits (*S. lycopersicum* Will.) LITTH-778, LITTH-784, LITTH-786, LITTH-788, and LITTH-790, in which the shelf life was extended by 10 days compared to the pollinated fruits [41]. The higher content of gibberellins in parthenocarpic tissues delays the cell wall degradation and, as a consequence, prolongs the fruit’s shelf life [42,43]. It has been reported that in tomato, after the application of prohexadione-Ca (GA biosynthesis inhibitor), the shelf life of the fruit decreases by four days due to the overexpression of the *TFM7-SlGA2ox1* transgene that reduces GA biosynthesis [44]. In pea (*Pisum sativum*), the expression of the *PsGA3ox1* gene (GA3-oxidase-1) in the pericarp has been associated with the maintenance of cell wall integrity and the enlargement of exocarp parenchyma cells through the induction of genes involved in the biosynthesis of cellulose, xylan, and lignin [45,46]. This structural reinforcement contributes to an extended shelf life of the fruit, as it preserves the integrity of the pericarp cell walls.

Parthenocarpy also decreased the weight loss in the fruits, being more marked in the CP40-P. These data are important since losses greater than 0.8% per day in cactus pear cause irreversible alterations in commercial quality [47]. The lowest weight loss has also been reported in “Flame Seedless” grapes treated with 25 mg dm^−3^ of GA_3_ and 10 mg dm^−3^ of CPPU; parthenocarpic fruits had lower weight loss (3.7%) compared to control fruits (7.4%) after 7 days of storage [48]. Stomatal development is determined at anthesis and remains constant, but as the fruit grows, stomatal frequency decreases due to expansion [49]. Pollination induces the expression of the *WOX13* gene, which promotes cell division and stomatal development. In contrast, parthenocarpic fruits exhibit reduced stomatal formation because the cell division stops during stage II, and growth depends on cell elongation [50,51]. Consequently, parthenocarpic fruits develop a lower stomatal density, which is associated with reduced water loss. Anatomical analyses revealed that their pericarpel is composed of fewer, yet larger, parenchyma cells.

In the varieties analyzed in this study, the total soluble solids (TSS) content was comparable to that reported for *Opuntia ficus-indica* cv. “Orito” (14.9 °Brix), with lower acidity (0.9%) [52], then the TSS/TA ratio in our fruits was notably higher (ranging from 48 to 75) compared to cv. “Orito” (TSS/TA = 17). In *O. ficus-indica* L. (Mill), glucose and fructose have been identified as the predominant sugars [7]. Changes in fruit firmness during storage are influenced by dehydration, modifications in the middle lamella, and degradation of cell wall components, processes regulated by phytohormones [53]. These changes vary depending on the fruit type, cultivar, and maturity. In parthenocarpic fruits of *Annona squamosa* treated with 1 g L^−1^ GA_3_ at 0, 7, 21, and 35 days after anthesis, lower firmness was recorded at harvest compared to pollinated fruits (2.83 vs. 8.41 N). This was associated with the presence of larger mesocarp cells with thicker cell walls, but also with wider spaces in the middle lamella. Furthermore, parthenocarpic fruits showed pronounced loosening of the cell wall, linked to the proliferation of secretory vesicles, dictyosomes, and mitochondria. In contrast, pollinated fruits had a higher number of irregularly shaped cells and less intercellular space, characteristics that contribute to greater firmness [54]. This anatomical variation may explain the lower firmness observed in the pericarpel of CP30-P fruits, where the cells are larger and arranged vertically.

Pectin methylesterase (PME) catalyzes the de-esterification of galactosyl methyl esters in pectin to form free carboxyl groups; this demethylation facilitates the activity of polygalacturonase (PG), weakening cell wall integrity and reducing intercellular adhesion, key factors in fruit softening [55,56]. PG is the most important enzyme in pectin degradation during ripening, and PME-mediated demethylation is a prerequisite for its action [57]. In the fruits analyzed, higher PG activity was observed in the pericarpel of CP30 pollinated fruits, which may contribute to their reduced shelf life and thinner pericarpel, these fruits also showed greater susceptibility to pathogen attack; in the other hand, the pulp of CP40-P the higher activity of PG and PME resulted in lower firmness. In strawberries, increased PG activity has been positively correlated with *Botrytis cinerea* infection, as PG-mediated degradation of the cell wall facilitates fungal invasion [58].

Antioxidant enzyme activity, specifically catalase (CAT) and ascorbate peroxidase (APX), was consistently higher in the pericarpel of parthenocarpic fruits throughout storage. A similar trend was reported in parthenocarpic “Shixia” longan fruits, where CAT activity reached 390 U g^−1^ FW by day six of storage, compared to 220 U g^−1^ FW in control fruits. For APX, activity reached 140 U g^−1^ FW in parthenocarpic fruits after 12 days, while in control fruits it was 90 U g^−1^ FW. These enzymes play essential roles in reducing H_2_O_2_ levels in plant tissues and are the key to mitigating oxidative stress. Elevated CAT and APX activity helps to protect membrane lipids from reactive oxygen species (ROS), particularly in tissues treated with growth regulators [59].

Cactus prickly pears have different pigments that give them their attractive color. Among them are betalains, which are a class of nitrogenous pigments that are synthesized from conjugations of betalamic acid with other structures, being betacyanins (red-violet) and betaxanthins (yellow-orange). Betalains are cancer preventive agents with properties that can protect human cells from oxidative stress and detoxify the body. These pigments are present in cactus pear, beet (*Beta vulgaris*), pitahaya (*Hylocereus undatus* and *H. polyrhizus*), chard, and amaranth [60]. The betacyanin content in our four varieties was higher compared to the other reports of prickly pear varieties, such as CP1 (red) and CP4 (yellow), with 1–7 mg 100 g^−1^ FW, and the betaxanthin content of 1.0–5.5 mg 100 g^−1^ FW [61]. In addition, the content of betacyanins in CP30 yellow varieties is up to 90% higher than that of CP40 red fruits, but with no differences in betaxanthins between varieties.

Ascorbic acid is unstable and degrades under the effects of light, oxygen, water activity, high temperatures, and pH [62]. The range of ascorbic acid content in the pericarpel and pulp of *O. ficus indica* is 50–70 mg 100 g^−1^ FW, more stable than that reported in *O. robusta* (15 and 92 mg 100 g^−1^ FW) [63]. Therefore, these varieties are among those reported for *O*. *ficus-indica* since the content is around 57 mg 100 g^−1^ FW in pericarpel and 62 mg 100 g^−1^ FW in pulp. The ascorbic acid content is considerably higher than that reported in pitahaya (*H. undatus* and *H. ocamponis*) with 5.0 mg and 10.1 AAE 100 g^−1^, respectively [64].

Phenolic compounds have important antioxidant activity, which can contribute to preventing human degenerative diseases [65]. In *O. fícus indica* cv. Sicilianos reported that the concentration of total phenols in fruits is higher in the pericarpel than in pulp [66]. The edible pericarpel of CP30-P fruits becomes relevant, with the highest content of phenolic compounds. In parthenocarpic tomato, the concentration of phenols is up to 20% higher than in pollinated fruits [41]. Regarding antioxidant activity, the prickly pear cultivars “Shafawi” (red) and “Toti” (yellow) showed a radical elimination of 35% in the pulp [67], lower compared with our analyzed varieties, which were above 60%. The flavonoid content in the pericarpel and pulp of the parthenocarpic fruits was higher compared with the pollinated fruits. This is similar to what was observed in parthenocarpic fruits of “Shixia” longan treated with growth regulators; these fruits presented up to 50% more flavonoids on days 9 and 12 of storage compared to pollinated fruits. This may be due to hormonal stimulation by gibberellins and cytokinins. Exogenous growth regulators such as GA_3_, GA_4_+_7_, BA, or CPPU activate the phenylpropanoid pathway. This is a precursor of flavonoids by inducing the expression of key genes such as PAL (phenylalanine ammonia-lyase), CHS (chalcone synthase), and FLS (flavonol synthase), leading to a greater accumulation of flavonoids, which function as antioxidants [59].

Auxins are naturally present in plants and play key roles in numerous biological processes triggered by environmental stimuli, including cell signaling, cell cycle regulation, endocytosis, embryogenesis, organogenesis, and growth modulation [68]. The most common auxins include indole-3-acetic acid (IAA), indole-3-butyric acid (IBA), 4-chloroindole-3-acetic acid (4-Cl-IAA), and phenylacetic acid (PAA), with IAA being the most abundant. IAA and IBA are also involved in fruit ripening, where they act as negative regulators by reducing reactive oxygen species (ROS), thereby limiting oxidative stress, maintaining membrane integrity, and preventing lipid peroxidation [69]. In the analyzed fruits, auxin levels were up to 50% higher in parthenocarpic fruits, which also showed a longer shelf life. Similar results were reported in two pepper cultivars (*Capsicum annuum* L.), “TK” and “CG”, stored at 2 °C for 21 days. In cultivar “TK”, indole-3-acetic acid (IAA) levels increased markedly from 10 to 80 µg kg^−1^ after just 3 days of storage, whereas in “CG”, IAA content remained constant at 10 µg kg^−1^. After 21 days of storage, “TK” fruits maintained a better visual appearance and no signs of chilling injury, in contrast to “CG” fruits, which exhibited severe damage [70].

Gibberellins are synthesized in buds, leaves, and fruits. They are commonly used to induce parthenocarpy and, along with other phytohormones, play a central role in fruit size regulation [71]. Parthenocarpic fruits typically exhibit higher levels of gibberellins, contributing to fruit growth and development [72]. In *Capsicum annuum* L. cv. PKM-1, fruits treated with GA_3_ had a shelf life of 30 days, compared to 15 days in control fruits [73]. In *Pisum sativum*, the *PsGA3ox1* (GA_3_-oxidase-1) gene expressed in the pericarp has been associated with cell wall maintenance and enlargement of exocarp parenchyma cells [46]. This gene also induces the expression of MYB, NAC, CESA, and PAL, which are involved in the biosynthesis of cellulose, xylan, and lignin [45].

Abscisic acid (ABA), derived from the carotenoid biosynthesis pathway, originates from C_40_-cis-epoxycarotenoids through the action of 9-cis-epoxycarotenoid dioxygenase (NCED), producing xanthoxin, the C_15_ precursor of ABA. ABA plays a central role in the ripening of non-climacteric fruits. Its antagonistic relationship with gibberellins is well documented, influencing various developmental and stress-response processes. Biosynthetically, ABA, gibberellins, phytol, and carotenoids share a common precursor—geranylgeranyl pyrophosphate (GGPP)—leading to metabolic competition [74]. However, recent studies also report a simultaneous and specific regulation of hormonal biosynthesis in parthenocarpic fruits, rather than a direct competition by GGPP, as observed in this study, where ABA levels increased in CP40-P fruit. In tomato, a coordinated regulation of multiple hormones (ABA, GA, auxins) and transcription factors was observed, indicating that not always exists an antagonic role between hormones [16]; also in melon, GA induction by *GA20ox* did not reduced ABA, suggesting an elevated effect of GGPP flux [75]. In non-climacteric fruits such as grapes, citrus, and strawberries, ABA is essential for maturation and exerts a strong influence on fruit physiology [76]. For example, in blueberries, fruits with the highest ABA content (0.6 µg g^−1^ FW) by day 8 of storage showed a firmness of 2 N and were considered unmarketable, while control fruits with lower ABA levels (0.2 µg g^−1^ FW) retained 3 N firmness and were acceptable for consumption [77]. A similar pattern was observed in this study: pollinated fruits had ABA levels approximately 50% higher than their parthenocarpic counterparts, and a notably shorter shelf life. This increase in growth regulator levels may suggest continued phytohormone biosynthesis in the fruit tissues.

## 5. Conclusions

Parthenocarpic prickly pear fruits of the CP30-P and CP40-P varieties have a longer shelf life than pollinated fruits, although they are smaller. Other characteristics of parthenocarpic fruits include a thicker cuticle and pericarpel with the presence of sclereids and hypodermis, and a lower stomatal frequency. No significant differences were observed between seeded and parthenocarpic fruits in terms of TSS, acidity, and sugar content. Parthenocarpic prickly pear fruits Parthenocarpic fruits have the advantage over pollinated ones in that they have almost imperceptible seeds, edible peel, and longer shelf life.

## Figures and Tables

**Table 1:** Weight loss of prickly pear fruits (*O. ficus-indica* L. (Mill) at 21 ± 1 °C and 61 ± 2% RH.

Storage (Days)	Varieties/Weight Loss (%)			
	CP30	CP30-P	CP40	CP40-P
14	9.7 ± 1.1	9.3 ± 2.3	6.2 ± 2.2	5.8 ± 0.9
16	--	9.4 ± 2.6	7.6 ± 3.0	6.2 ± 0.9
30	--	12.4 ± 2.8	--	9.7 ± 1.6
32	--	12.5 ± 1.9	--	--

CP30: Pollinated fruit, CP30-P: Parthenocarpic, CP40: Pollinated, CP40-P: Parthenocarpic. -- Shelf life was over, (*n* = 10 ± SD).

**Table 2:** Concentration of total phenols (day 0 and 10 of storage) and antioxidant activity (day 0 of storage) of prickly pear fruits (*O. ficus-indica* L. (Mill)) at 21 ± 1 °C and 61 ± 2% RH.

Varieties	Total Phenols (mg GA 100 g^−1^ FW)				Antioxidant Activity (% RSC)	
	0		10			
	Per	Pul	Per	Pul	Per	Pul
CP30	97.3 ± 4.4 a *	96.2 ± 15.2	20.9 ±2.9 b	14.9 ± 0.5	69.3 ± 8.2	75.3 ± 12.5
CP30-P	81.2 ±12.9 b	78.2 ± 13.5	52.0 ± 10.4 a	14.4 ± 1.5	58.2 ± 9.4	67.4 ± 12.1
CP40	91.2 ± 32.1	90.2 ± 21.5	16.7 ± 0.5	15.9 ± 1.3 a	82.3 ± 14.1	75.5 ± 14.5
CP40-P	82.1 ± 5.0	81.2 ± 8.2	14.9 ± 2.2	13.4 ± 1.2 b	77.0 ± 9.4	74 ± 9.8

* Values with different letters indicate statistically significant differences (Tukey, *p* ≤ 0.05) between pollinated and parthenocarpic fruits. Per: Pericarpel, Pul: Pulp. CP30: Pollinated fruits, CP30-P: Parthenocarpic, CP40: Pollinated fruits, CP40-P: Parthenocarpic (*n* = 5 ± SD).

**Table 3:** Concentration of total flavonoids (day 0 and 10 of storage) and antioxidant activity (day 0 of storage) of prickly pear fruits (*O. ficus-indica* L. (Mill) at 21 ± 1 °C and 61 ± 2% RH.

Varieties	Total Flavonoid (mg QE 100 g^−1^ DW)			
	0		10	
	Per	Pul	Per	Pul
CP30	94.7 ± 12.4 b *	26.3 ± 2.7	116.8 ± 1.7 b	28.6 ± 3.0
CP30-P	130.6 ± 11.6 a	22.9 ± 1.6	145.2 ± 0.7 a	32.7 ± 4.5
CP40	124.0 ± 8.5 b	43.5 ± 2.9 a	139.7 ± 4.8 b	46.6 ± 1.7 b
CP40-P	145.6 ± 2.3 a	32.3 ± 2.9 b	145.6 ± 3.1 a	53.3 ± 5.5 a

* Values with different letters indicate statistically significant differences (Tukey, *p* ≤ 0.05) between non-parthenocarpic and parthenocarpic varieties. Per: Pericarpel, Pul: Pulp. CP30: Pollinated; CP30-P: Parthenocarpic, CP40: Pollinated, CP40-P: Parthenocarpic (*n* = 5 ± SD).

**Table 4:** Concentration of pigments in prickly pear fruits (*O. ficus-indica* L. (Mill)), on day 0 of storage at 21 ± 1 °C and 61 ± 2% RH.

Varieties	Chlorophyll (mg 100 g^−1^ FW)		Carotenoids (mg 100 g^−1^ FW)	
	Per	Pul	Per	Pul
CP30	2.0 ± 0.6 *	0.7 ± 0.1	7.0 ± 1.0	5.0 ± 0.8 a
CP30-P	2.0 ± 0.4	0.9 ± 0.2	5.0 ± 1.0	3.0 ± 0.4 b
CP40	3.0 ± 0.1	0.6 ± 0.1	14.6 ± 1.0 a	6.0 ± 0.2
CP40-P	3.0 ± 0.4	0.9 ± 0.1	9.6 ± 1.0 b	4.0 ± 0.1

* Values with different letters indicate statistically significant differences (Tukey, *p* ≤ 0.05) between non-parthenocarpic and parthenocarpic varieties. Per: Pericarpel. Pul: Pulp. CP30: Pollinated; CP30-P: Parthenocarpic; CP40: Pollinated; CP40-P: Parthenocarpic (*n* = 5 ± SD).

**Table 5:** Concentration of betalains in prickly pear fruits (*O. ficus-indica* L. (Mill)) on day 0 of storage at 21 ± 1 °C and 61 ± 2% RH.

Varieties	Betacyanins (mg 100 g^−1^ FW)		Betaxanthins (mg 100 g^−1^ FW)	
	Per	Pul	Per	Pul
CP30	25.9 ± 12.3 *	13.4 ± 2.0 b	16.8 ± 1.3 a	9.6 ± 1.1 b
CP30-P	24.1 ± 12.0	18.4 ± 2.3 a	15.0 ± 0.7 b	14.3 ± 4.4 a
CP40	2.3 ± 0.6	1.5 ± 0.3	15.2 ± 2.9	14.5 ± 1.1
CP40-P	2.3 ± 0.5	1.6 ± 0.2	17.2 ± 3.9	16.0 ± 1.1

* Values with different letters indicate statistically significant differences (Tukey, *p* ≤ 0.05) between non-parthenocarpic and parthenocarpic varieties. Per: Pericarpel, Pul: Pulp. CP30: Pollinated, CP30-P: Parthenocarpic, CP40: Pollinated, CP40-P: Parthenocarpic (*n* = 5 ± SD).

**Table 6:** Catalase (CAT) and ascorbate peroxidase (APX) activity of prickly pear fruits (*O. ficus-indica* L. (Mill)). On days 0, 15, and 30 of storage at 21 ± 1 °C and 61 ± 2% RH.

Assessment Day/Varieties	CAT (U g^−1^ FW)	APX (U g^−1^ FW)
0 days of storage		
CP30	21.0 ± 3.2 *	35.8 ± 2.7 a
CP30-P	22.1 ± 2.6	31.4 ± 2.2 b
CP40	24.4 ± 4.5 a	40.7 ± 2.9
CP40-P	19.4 ± 2.5 b	38.7 ± 5.1
15 days of storage		
CP30	878.1 ± 64.3 b	235.6 ± 38.5 b
CP30-P	1495.0 ± 72.4 a	300.1 ± 60.1 a
CP40	657.9 ± 29.3 b	401.4 ± 48.3 b
CP40-P	1126.9 ± 36.5 a	420.4 ± 21.7 a
30 days of storage		
CP30-P	948.0 ± 55.3	243.9 ± 28.5
CP40-P	1029.6 ± 91.7	315.6 ± 16.8

* Values with different letters indicate statistically significant differences (Tukey, *p* ≤ 0.05). CP30: Pollinated; CP30-P: Parthenocarpic, CP40: Pollinated, CP40-P: Parthenocarpic (*n* = 5 ± SD).

## Data Availability

The original contributions presented in this study are included in the article. Further inquiries can be directed to the corresponding author.
